# Behavioral and psychosocial predictors of depression in Bangladeshi medical students: a cross-sectional study

**DOI:** 10.12688/f1000research.122927.1

**Published:** 2022-07-05

**Authors:** Md Rizwanul Karim, Helal Uddin Ahmed, Shahnaz Akhter

**Affiliations:** 1 Department of Community Medicine, Rajshahi Medical College (RMC), Rajshahi, 6000, Bangladesh; 2 Department of Child Adolescent & Family Psychiatry, National Institute of Mental Health (NIMH), Shyamoli, Dhaka, 1207, Bangladesh; 3 Department of Gynae and obstetrics, Combined Military Hospital, Jalalabad, Sylhet Cantonment, Sylhet, 3107, Bangladesh

**Keywords:** Depression, anxiety, Perceived stress, sleep quality, Facebook addiction

## Abstract

Background: Depression, stress, and anxiety were found in a large number of medical undergraduate students, indicating a neglected aspect of their psychology that required immediate attention. The goal of this study was to find out the prevalence of depression among medical students, as well as potential psychosocial and behavioral predictors for depression.

Methods: This cross-sectional study was conducted from July to November 2021 among 840 randomly selected medical students from four medical colleges using stratified random sampling. Data were collected using a semi-structured, self-administered questionnaire and were analyzed through the SPSS v.23 software. Multiple regression was performed to assess the effect of several behavioral and psychosocial factors on depression.

Results: Among the 840 study participants, 55.7% (n= 468) were female and 44.3% (n= 372) were male. According to the data, the prevalence of depression, anxiety, perceived stress among medical students was found to be 28.8%, 65% and 85% respectively. A strong link was found between depression and anxiety, stress, poor sleep quality, poor academic performance, and a negative social and romantic relationship status.

Conclusions: A significant number of medical students are depressed. In order to prevent and treat depression, medical students should be screened for depression and its associated factors.

## Introduction

Depression affects about 5.0% of the world’s adult population. There are about 280 million people in the world suffering from depression. ^1^ Recurrent and severe depression can negatively affect a person’s performance at work, at school and life at home. At its worst, depression may lead to suicide. The World Health Organization (WHO) lists suicide as the fourth most common cause of death among 15-29-year-olds. According to the national mental health survey of Bangladesh provisional fact sheet 2018-19, the prevalence of depression was 6.7% (5.8-7.6). ^2^

A medical student’s mental health is generally similar to or better than that of the general population before they enter the medical school. ^3^^–^^7^ But among medical students who have begun their course, depression is one of the most common mental health issues due to the intensity of the training. ^8^ Undergraduate medical education requires intense study and training for five to six years. Because of advancing knowledge and evolving therapies, the curricular objectives are constantly evolving. The learning period should prepare medical students to deal independently with lifelong professional challenges by acquiring sufficient professional knowledge, skills, and attitudes. Students’ physical and mental health could be adversely affected by the demands of studying and training. As a consequence, a number of medical students are reported to suffer from depression, anxiety, and stress. ^9^^–^^11^

Medical students are subjected to various academic and psychosocial stressors that are thought to be typical of their environment. ^7^^,^^12^ As medical students, they deal with workload, ^12^^–^^14^ academic pressure, ^15^ the pressure to demonstrate competence as a clinician, ^16^ sleep deprivation, ^13^ peer competition, ^13^ fear of failure, ^13^ the death and suffering of patients, ^17^ student abuse, ^18^ financial problems, ^13^^,^^14^ etc. Several systematic reviews and meta-analyses showed that medical students around the world suffer from depression at a rate of 28%, and that suicidal ideation occurs at a rate of 11.1%. ^19^^–^^21^ In the world, one in three medical students are depressed, ^19^ a rate much higher than the general population (3.9–6.6%) ^22^^,^^23^ and nonmedical students (19% in men, 22% in women). ^24^ In addition, first-year residents reported an increased level of depression. ^25^ Chronic sleep deprivation, ^25^ perceived stress, and anxiety have all been demonstrated to be important predictors of depression. ^26^ Identifying risk factors for depression among medical students should be a priority because depression among this group can lead to low quality of life, dropping out, ^27^ and eventually suicidal ideation.

Internet Addiction (IA) was found to be linked to depression and academic issues in a Thai study and the odds of depression in the possible IA group were 1.58 times higher than in the normal Internet use group (95%CI: 1.04–2.38). ^28^

Around 40% of Bangladeshi students were found to be at danger of Facebook addiction. Being of single status, a lack of physical activity, sleep disruption, Facebook use time, and depression symptoms have all been linked to Facebook addiction. ^29^ Internet Addiction and depression have previously been linked in adults, ^30^ adolescents, ^31^^–^^33^ and college students. ^34^ In a systematic review of comorbid psychopathology in pathological Internet use, 75% of the papers examined indicated a substantial link between pathological Internet use and depression. ^35^ When compared to other mental comorbidities, depression exhibited the strongest connection to pathological Internet use. ^34^ The prevalence of depression was consistently higher in the IA group than in the control group, according to a meta-analysis of five trials. ^36^

Medical students have been shown to have a high level of psychological morbidity, ^37^^–^^42^ and they experience more psychological distress than the general population. ^40^ Depression, stress, and anxiety were observed in a considerable number of medical undergraduate students, indicating a neglected element of their psychology that needed prompt care. ^43^ Otherwise, students’ distress can have a negative impact on professional development and academic performance, as well as contribute to academic dishonesty, substance abuse, and medical school attrition. ^40^ According to earlier research on medical school graduates, stress has a detrimental impact on patient care quality, patient safety, ^44^ and professionalism. ^45^

Both physical and mental wellness require adequate and high-quality sleep, ^46^^,^^47^ whereas poor sleep quality or insufficient sleep are linked to unhealthy lifestyle habits such as internet use. Excessive internet use is, in fact, a leading predictor of poor sleep quality and workplace negligence. ^48^ Furthermore, during health emergencies, studies show that the general population experiences increased fear, anxiety, and stress, and that these unfavorable emotional and mental health situations negatively affect the public’s sleep quality, particularly university students. ^49^

Alarmingly, many Bangladeshi medical students have recently committed suicide. ^50^^,^^51^ Stress, depression, and anxiety among undergraduate students are underdiagnosed in more than half of instances, according to previous studies in other countries. Furthermore, they are frequently undertreated, resulting in increased psychological morbidity, which has a negative impact on their job and personal lives. ^52^

The purpose of this study was to determine the prevalence of depression among medical students at Public Medical Colleges as well as implicit depression predictors such as anxiety, perceived stress, internet addiction, Facebook addiction, sleep quality, sociodemographic and behavioral factors.

### Ethical statement

Ethical approval was obtained from the Bangladesh Medical Research Council (BMRC) Ethical Review Committee (BMRC/HPNSP-Research IRB 2020-2021 I 320). All ethical issues related to this study were carefully addressed in accordance with the Helsinki Declaration including their privacy, confidentiality and anonymity. There was no invasive procedure or private issue in the study, and no drug was tested. Before starting the data collection process, a brief explanation of the study’s goals and objectives was given to the respondents. Their written informed consent were then obtained in a separate consent form that was attached to the main questionnaire. They were informed of their complete right to participate or decline in the study. A semi-structured self-administered questionnaire was used to collect data. Respondents were assured that the information provided by them e.g., their names or anything which could identify them, would be kept confidential and anonymous and the anonymized data and or results of the study will only be disseminated and published for public interest.

## Methods

### Study design

This cross-sectional study was conducted from July to November 2021, among students from four public medical colleges chosen at random from among 37 public medical colleges in Bangladesh.

### Study population

The sample size was determined based on a 95% confidence interval and a 5% sampling error. The required sample size to estimate a true prevalence of depression was computed using Epitools. Assuming the prevalence of depression among medical students of 39.1 ^53^ and sensitivity 74% and specificity 91% for PHQ9, ^54^ the estimated sample size was 838. Using two-step stratified random sampling we enrolled 840 medical students from a total of 5000 medical students (4*1250) from four selected medical colleges. First, a request letter explaining the study’s objectives was sent to the principal offices of the corresponding medical colleges. The research team then organized a presentation and question-and-answer session on the study protocol for each year’s students in collaboration with the respective medical college administration. In the final step, 42 students were chosen at random from each year, ranging from the first to the fifth year, for a total of 210 students from each medical college.

### Data collection

The pretested Bengali version of the questionnaire was translated and back-translated by two independent bilingual translators to check the consistencies avoiding response bias. The selected respondents were given the Bengali version of the questionnaire and asked to complete it in their own time within a three-day window. It took them approximately twenty-five minutes to complete the self-reported survey. Before implementing the survey, formal permission from the IRB was obtained as well as written consent from the participants. After checking for inconsistencies and missing values, all 840 participants were found to have completed the entire survey questionnaire and the data were entered into the SPSS v-23 software for further analysis.

### Measures

The questionnaire included a total of 122 questions and was divided into eight sections, as follows:

**1. Socio-demographic variables**

This section included several questions on socio-demographic variables, including age, gender, permanent residence (city or village area), relationship status (single or in a relationship), Parental education, average monthly income, type and place of residence, religion, etc.

**2. Internet use and Facebook use related variables**

The variables in this section were: device used, type of network, time since they started using the internet, primary purpose of internet use, average daily use, average monthly expenditure for internet use, total ID, fake ID, effect of internet on financial status, academic status, effect on relationship with friends and family, effect on romantic relationship, number of friends, followers, average number of posts, average comment and reaction per FB post, self-measures to limit internet use.

**3. Chen Internet Addiction Scale CIAS**

The CIAS is a four-point, 26-item self-reported scale that assesses five dimensions of Internet-related behavioral attributes, including compulsive use, withdrawal, tolerance, interpersonal relationship problems, and health/time management issues. ^55^ The Chen Internet Addiction Scale has a total score range of 26 to 84, with higher CIAS scores indicating more severe Internet Addiction. Internal reliability for the scale and its subscales in the initial study ranged from 0.79 to 0.93. A previous literature argued that the diagnostic cut-off point (63/64) gave CIAS the best diagnostic accuracy, Cohen Kappa, and DOR and using this point, more than 80% of cases can be correctly classified. ^56^

With the exception of the Chen Internet Addiction Scale CIAS, which was used unchanged to measure internet addiction among medical students, all of the scales used in the study had previously been validated in Bengali. For this purpose, a pilot survey was conducted among 260 medical students in two public medical colleges other than the four medical colleges chosen for the study sample; 26 medical students were chosen at random from each session’s attendance registration (first year to fifth year) from two medical colleges. The study purpose was communicated to them prior to any interviews, and their informed consent was obtained. The items and dimensions of the original CIAS scale items used in this study were described and rationalized using factor analysis, which supports the tool’s use in this study.

We used our study data to conduct principal component analysis (PCA) on the 26 items of the Chen Internet Addiction Scale to support the pilot survey. The suitability of the data for factor analysis was determined prior to performing PCA. The correlation matrix revealed the presence of all coefficients of.36 and higher. The Kaiser-Meyer Olkin value was .94, which exceeded the recommended value of .6, ^57^ and the Bartlett’s Test of Sphericity ^58^ reached statistical significance, indicating that the correlation matrix was factorable. The presence of five components with eigen values greater than one was revealed by principal component analysis. and the five-component solution explained a total of 50.23% of the variance with Component 1 to Component 5 contributing, 12.04%, 11.07%, 10.94%, 10.21 and 5.97% of the variance respectively. An inspection of the scree plot revealed a clear break after the fifth component. Using Cattell’s ^59^ scree test, it was decided to retain five components for further investigation.

To aid in the interpretation of these five components, varimax rotation was performed. The rotated solution (presented in Table 1) revealed the presence of simple structure (Thurstone, 1947), with components showing several strong loadings, and all variables loading substantially on only one component. The interpretation of the five components was consistent with the type of items; seven items loaded onto Factor 1, which are related to compulsive use. Factor 2 comprised of five items reflect health-time conflict, factor 3 consists of six items related to behavioral and social distraction, factor 4 consists of five items which indicate tolerance and finally factor 5 consists of three items related to withdrawal ( Table 1).

**Table 1.** Factor analysis: Chen internet addiction scale (26 items) five factor solution.

Rotated component matrix ^a^					
	Component				
	1	2	3	4	5
I was told more than once that I spend too much time online.	.737				
I find that I have been spending longer and longer periods of time online.	.676				
I have tried to spend less time online but have been unsuccessful.	.603				
I stay online for longer periods of time than intended.	.588				
I have increased substantially the amount of time I spend online.	.562				
Although using the Internet has negatively affected my relationships, the amount of time I spend online has not decreased.	.453				
My interactions with family members have decreased as a result of Internet use.	.360				
I feel tired during the day because of using the Internet late at night.		.667			
I find myself going online instead of spending time with friends.		.652			
I fail to have meals on time because of using the Internet.		.599			
More than once, I have slept less than four hours due to being online.		.591			
Suffering the Internet has negatively affected my physical health.		.507			
I need to spend an increasing amount of time online to achieve the same satisfaction as before.			.661		
I fail to control the impulse to log on.			.619		
I make it a habit to sleep less so that more time can be spent online.			.615		
I get backaches or other physical discomfort from spending time surfing the net.			.544		
I feel distressed or down when I stop using the Internet for a certain period of time.			.494		
Going online has negatively affected my school work or job performance.			.382		
My life would be joyless without the Internet.				.624	
I feel like I am missing something if I don't go online for a certain period of time.				.525	
My recreational activities have decreased as a result of Internet use.				.523	
I fail to control the impulse to go back online after logging off for other work.				.518	
Going online is the first thought I have when I wake up each morning.				.457	
I feel energized online.					.784
I feel restless and irritable when the Internet is disconnected or unavailable.					.533
I feel uneasy once I stop going online for a certain period of time.					.435

Extraction Method: Principal Component Analysis.

Rotation Method: Varimax with Kaiser Normalization. Cronbach's Alpha = .897.

^a^ Rotation converged in 12 iterations.

The internal consistency of the whole Chen Internet Addiction Scale (CIAS) is.90 (Chronbach’s alpha). The split half correlation (> .80) and Guttman correlation coefficient (> .80) also support the rationality of use of 26 items Chen Internet Addiction Scale to measure internet addiction among Bangladeshi medical students (see Table 2).

**Table 2.** Reliability Statistics for Chen Internet Addiction Scale (CIAS).

Cronbach's Alpha	Part 1	Value	.817
		N of items	13
	Part 2	Value	.834
		N of items	13
	Total N of Items		26
Guttman Split-Half Coefficient			.846

**4. Bergen Facebook Addiction Scale (BFAS)**

The Bergen Facebook Addiction Scale ^60^ is 6 items measured with a five-point Likert-type scale (1 = very rarely, 2 = rarely, 3 = sometimes, 4 = often, 5 = very often). This scale is concerned with experiences of six core elements of addiction i.e., salience, tolerance, mood modification, conflict, relapse, and withdrawal during the past year related to Facebook use. The Cronbach’s Alpha of this measure in the original study was 0.83. Item-total correlations ranged from 0.60 to 0.73 and the test-retest reliability was 0.82 as reported by authors.

Bangla BFAS had a sufficient level of reliability and validity and this measure could be applicable in Bangladeshi culture for screening out Facebook addiction. ^61^

**5. Perceived Stress Scale PSS10 B**

The PSS-10 measures the degree to which one perceives aspects of one’s life as uncontrollable, unpredictable, and overloading. ^62^ Participants are asked to respond to each question on a five-point Likert scale ranging from 0 (never) to 4 (very often), indicating how often they have felt or thought a certain way within the past month. Scores range from 0 to 40, with higher composite scores indicative of greater perceived stress. The PSS10 possesses adequate internal reliability. ^62^

The original English 10-item version of PSS ^62^ was translated into Bangla by different researchers. ^63^^–^^65^ One of the authors observed a significant correlation r = .90, p < .01 between the PSS-10-B (Bengali translated and adapted version of PSS10) ^64^ with the original English version of PSS-10. ^65^ Test-retest reliability of the Bangla adaptation was high over a period of two weeks, r = .94, p < .01, and indicated that the Bangla PSS-10 scale can be used to measure perceived stress of Bangladeshi people. ^65^

**6. Patient Health Questionnaire PHQ9**

The Patient Health Questionnaire (PHQ-9) is a nine-item self-administered scale developed to diagnose the presence and severity of depressive symptoms during over two weeks. The PHQ-9 total score ranges from 0 to 27, because each of the 9 items can be scored from 0 (“not at all”) to 3 (“nearly every day”). Easy-to-remember cut-points of 5, 10, 15, and 20 represent the thresholds for mild, moderate, moderately severe, and severe depression, respectively. If only one screening cut-point is used, researchers currently recommend a PHQ-9 score of 10 or higher, which has an 88 percent sensitivity for major depression, an 88 percent specificity, and a positive likelihood ratio of 7.1. ^66^

The Bengali translated version of PHQ9 showed good reliability; Cronbach’s alpha 0.837, gender-wise 0.839 for males and 0.841 for females; and the Spearman-Brown Coefficient is 0.855, and the Guttman Split-half coefficient is 0.848, which indicate the high Split-half reliability as well. ^67^

**7. Generalized Anxiety Disorder GAD7**

The GAD-7 is a self-administered seven-item instrument used as a screening tool for generalized anxiety disorder. Response options for each item range from 0 to 3 on a four-point Likert-scale (0 = not at all, 1 = several days, 2 = more than half the days and 3 = nearly every day). Adding the scores of all seven items provide the GAD-7 total score ranging from 0 to 21. Several validation studies have detected cut-points of ≥5, ≥10 and ≥15 based on receiver operating characteristics analysis for GAD-7, standing for mild, moderate and severe anxiety levels, respectively. ^68^

In Bangladesh, previous studies revealed good internal consistency of GAD-7 (Cronbach’s α = 0.87) ^69^ and good convergent validity of GAD-7 with two other scales, PHQ-9 and PHQ-ADS. ^70^

**8. Pittsburgh Sleep Quality Index (PSQI)**

The PSQI, one of the most widely used tools for evaluating sleep quality, is a parameter-based questionnaire that relies on self-reported responses. It was first developed by Buysse *et al.* in 1989. ^71^ The PSQI consists of 19 questions that are broken down into seven categories (subjective sleep quality, sleep latency, sleep duration, habitual sleep efficiency, use of sleep medication, and daytime dysfunction), all of which are weighted equally on a 0–3 scale to evaluate the quality of sleep over the previous month. ^71^ The seven component scores are then added together to produce a final PSQI sleep quality score that ranges from 0 to 21, with 0 being the best and 21 being the worst. Poorer sleep quality is indicated by a higher score. The validated Bangla version of the PSQI, also known as the Bengali Pittsburgh sleep quality index, was used in the current study to assess participants’ sleep quality (BPSQI). Poor sleep quality was defined as having a BPSQI score greater than 5. ^72^

### Statistical analysis

Data analysis was performed using SPSS statistical software version 23.0 (IBM Corp). Factor analysis and a reliability test were performed to assess the items and dimensions of CIAS in Bangladeshi medical students. Multiple regression was performed to assess the effect of several factors on the likelihood that respondents have depression according to PHQ-9 scale score. Analysis was conducted using depression as the dependent variable and a number of sociodemographic and behavioral factors as the independent variables, including age, sex, income, place of residence, housing status, type of family, physical activity, duration/cost and time of internet use, impact of personal social and academic and romantic life, internet addiction, Facebook addiction, perceived stress, anxiety, sleep quality, etc. The final results were presented with statistical significance, regression coefficients, and 95% confidence intervals for beta-coefficient for each of the predictors.

## Results

Among 840 study participants, 55.7% (n = 468) were female and 44.3% (n = 372) were male. The average age of the respondents was 21 years. 84% (n = 702) of the respondents were Muslim and 15% (n = 127) of the students were Hindu. In terms of their parents’ occupations, 31.5% (n = 265) of their fathers were businessmen, while 70% (n = 590) of their mothers stayed at home. One out of every ten respondents live alone, and seven percent of the medical students in the study sample were married. Three quarters of the respondents had personal income and 64%, n = 539 of the medical students resided in medical hostels.

**Figure 1.**
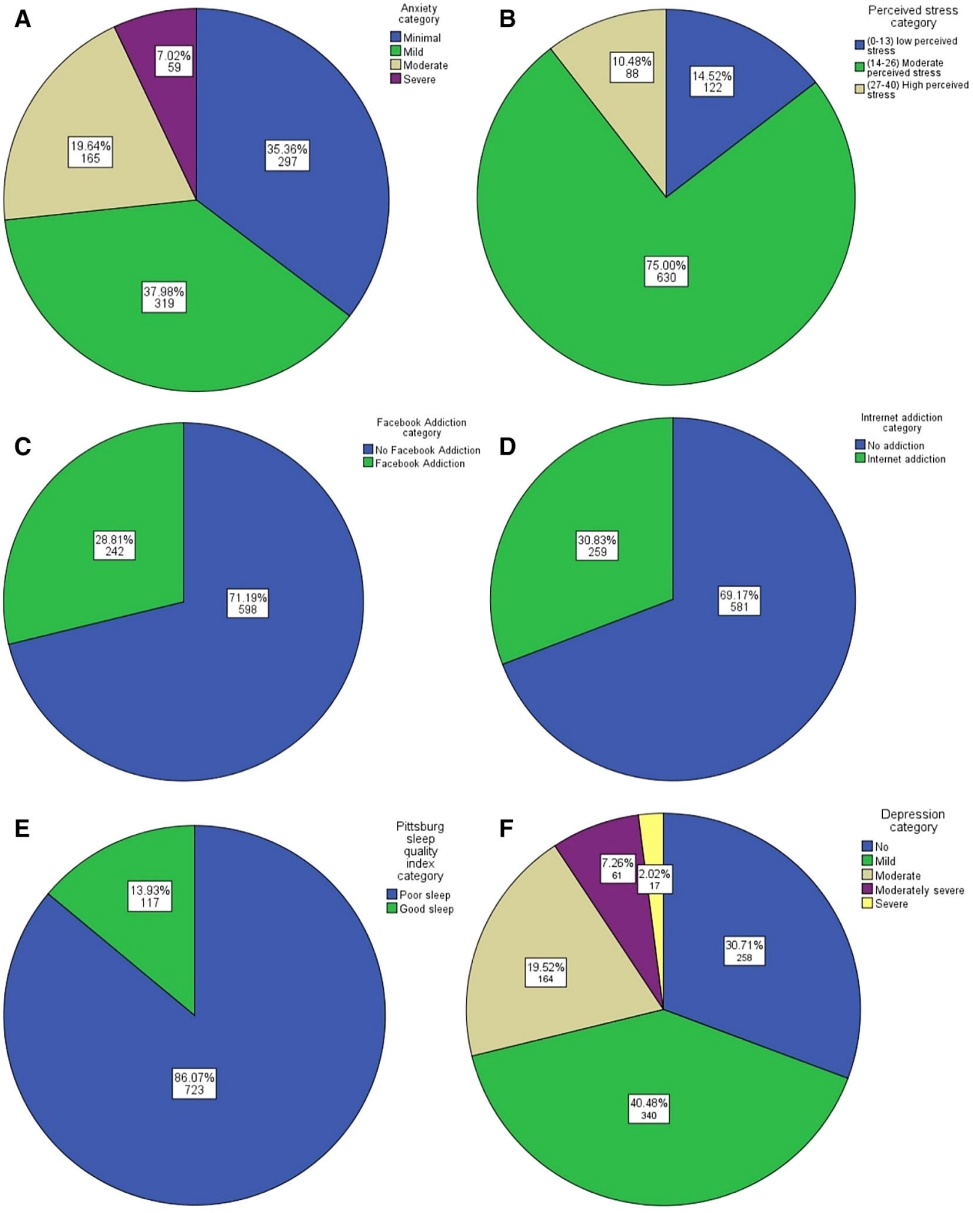
Distribution of anxiety, perceived stress, Facebook Addiction, Internet Addiction, sleep quality and depression among the respondents, N=840. A. Anxiety Levels of the respondents (GAD-7 scale cut-off). B. Perceived Stress Categories of the respondents (PSS10 cut-off). C. Facebook Addiction Categories of the respondents (BFAS cut-off). D. Internet addiction Categories of the respondents (CIAS cut-off). E. Sleep Quality among the respondents (PSQI cut-off). F. Depression categories of the respondents (PHQ-9 cut-off).

According to the data, 65% of respondents experienced mild to severe anxiety, ( Figure 1A) and 85% of medical students experienced moderate to high perceived stress ( Figure 1B). Alarmingly, 86% (n = 727) of the samples reported having fake Facebook ids and having used Facebook for an average of five years; Mean (SD) = 4.63 (1.88). Facebook Addiction and Internet Addiction were found in 28.8% ( Figure 1C) and 30.8% ( Figure 1D) of medical students, respectively. Eighty-six percent of the sample population slept poorly ( Figure 1E), and 28.8% of respondents suffered from moderate to severe depression ( Figure 1F). The full results of the analysis can be found under *Underlying data.* ^90^

Table 3 shows respondents with negative social, familial, and romantic relationships, as well as poor academic performance, were found to be more depressed, and the proportional differences between depression categories were shown to be statistically significant. [(χ ^2^ = 16.31, p = .000), (χ ^2^ = 35.007, p = .000), (χ ^2^ = 22.14, p = .000), (χ ^2^ = 29.54, p = .000)]. ^90^

**Table 3.** Effect of academic and relationship status on depression.

Academic and relationship status		PHQ9 depression category <10 and ≥10				
		No depression n (row %)	Depression n (row %)	Total n (column %)	χ ^2^	p
Friendships and social relationships	Average	304 (74.1)	106 (25.9)	410 (48.8)		
	Negative	76 (56.7)	58 (43.3)	134 (16.0)	16.31	.000
	Positive	218 (73.6)	78 (26.4)	296 (35.2)		
Family relationship	Average	418 (76.1)	131 (23.9)	549 (65.4)		
	Negative	95 (53.4)	83 (46.6)	178 (21.2)	35.007	.000
	Positive	85 (75.2)	28 (24.8)	113 (13.5)		
Romantic relationships	Average	494 (74)	174 (26)	668 (79.5)		
	Negative	39 (48.8)	41 (51.2)	80 (9.5)	22.14	.000
	Positive	65 (70.7)	27 (29.3)	92 (11)		
Academic performances	Average	248 (77.3)	73 (22.7)	321 (38.2)		
	Negative	187 (60.1)	124 (39.9)	311 (37.0)	29.54	.000
	Positive	163 (78.4)	45 (21.6)	208 (24.8)		

Facebook addiction was present among 28.8% of the study samples and was found to be significantly linked with depression (χ ^2^= 50.59, df 1, p = .000). All six domains of the Bergen Facebook Addiction Scale were also statistically significantly associated with depression (p ≤ .01) ( Table 4). Table 4 also shows that the presence of depression is 1.5 to 3 times more likely among the sample population who have features of Facebook addiction.

**Table 4.** Depression and different domains of Facebook addiction.

Bergen Facebook addiction scale domains		PHQ9 Total score (<10 and ≥10)		Total n (column %)	Crude OR with (95% CI)	χ ^2^	p
		No depression n (row %)	Depression n (row %)				
Salience	No = 0 = (<3)	398 (77.9)	113 (22.1)	511 (60.8)	2.27 (1.68, 3.08)	28.52	.000
	Yes = 1 = (≥3)	200 (60.8)	129 (39.2)	329 (39.2)			
Tolerance	No = 0 = (<3)	393 (74.3)	136 (25.7)	529 (63)	1.49 (1.10, 2.03)	6.70	.010
	Yes = 1 = (≥3)	205 (65.9)	106 (34.1)	311 (37)			
Mood modification	No = 0 = (<3)	384 (81.0)	90 (19.0)	474 (56.4)	3.03 (2.22, 4.13)	51.17	.000
	Yes = 1 = (≥3)	214 (58.5)	15 (41.5)2	366 (43.6)			
Relapse	No = 0 = (<3)	338 (77.3)	99 (22.7)	437 (52)	1.89 (1.39, 2.54)	16.83	.000
	Yes = 1 = (≥3)	260 (64.5)	143 (35.5)	403 (48)			
Withdrawal	No = 0 = (<3)	470 (77.2)	139 (22.8)	609 (72.5)	2.72 (1.97, 3.75)	38.68	.000
	Yes = 1 = (≥3)	128 (55.4)	103 (44.6)	231 (27.5)			
Conflict	No = 0 = (<3)	354 (78.8)	95 (21.2)	449 (53.5)	2.25 (1.65, 3.05)	27.53	.000
	Yes = 1 = (≥3)	244 (62.4)	147 (37.6)	391 (46.5)			
Overall Facebook addiction	No Facebook addiction (<4)	468 (78.3)	130 (21.7)	598 (71.2)	3.10 (2.25, 4.27)	50.59	.000
	Facebook addiction (≥4)	130 (53.7)	112 (46.3)	242 (28.8)			

Each of the Pittsburg Sleep Quality Index’s seven dimensions were statistically significantly associated with depression (p = .001). According to Table 5, 68% (28/41) of the sample population who have “very poor” perceived “overall sleep quality” have depressive symptoms.

**Table 5.** Depression and different aspects of sleep quality.

Pittsburg sleep quality index		PHQ9 Total score (<10 and ≥10)		Total n (column %)	χ ^2^	p
		No depression n (row %)	Depression n (row %)			
Duration of sleep	>7 (Better)	297 (72.1)	115 (27.9)	412 (49)	21.96	.000
	<7 and ≥6	194 (78.9)	52 (21.1)	246 (29.3)		
	<6 and ≥5	73 (61.3)	46 (38.7)	119 (14.2)		
	<5 (Worse)	34 (54.0)	29 (46.0)	63(7.5)		
Sleep disturbance	0 (Better)	52 (96.3)	2 (3.7)	54 (6.4)	98.16	.000
	≥1 and ≤9	473 (77.2)	140 (22.8)	613 (73)		
	>9 and ≤18	73 (42.2)	100 (57.8)	173 (20.6)		
Sleep latency	0 (Better)	171 (80.3)	42 (19.7)	213 (25.4)	50.48	.000
	≥1 and ≤2	275 (76.4)	85 (23.6)	361 (43)		
	≥3 and ≤4	120 (62.2)	73 (37.8)	193 (23)		
	≥5 and ≤6 (worse)	31 (42.5)	42 (57.5)	73 (8.7)		
Day dysfunction due to sleepiness	0 (Better)	15 (83.3)	3 (16.7)	18 (2.1)	29.43	.000
	≥1 and ≤2	116 (85.3)	20 (14.7)	136 (16.2)		
	≥3 and ≤4	293 (76.1)	92 (23.9)	407 (48.5)		
	≥5 and ≤6 (worse)	170 (60.9)	109 (39.1)	279 (33.2)		
Sleep efficiency	0 (Better)	455 (74)	160 (26)	616 (73.3)	14.26	.000
	≥1 and ≤2	95 (68.8)	43 (31.2)	138 (16.5)		
	≥3 and ≤4 (Worse)	47 (55.3)	38 (44.7)	86 (10.2)		
Overall sleep quality	Very good	13 (76.5)	4 (23.5)	17 (2)	36.32	.000
	Fairly good	512 (74.2)	178 (25.8)	691 (82.3)		
	Fairly bad	59 (64.8)	32 (35.2)	91 (20.8)		
	Very bad	13 (31.7)	28 (68.3)	41 (4.9)		
Need meds to sleep	Not during the past month	32 (82.1)	7 (17.9)	39 (4.6)	35.41	.000
	Less than once a week	360 (77.8)	103 (22.2)	463 (55.1)		
	Once or twice a week	137 (65.6)	72 (34.4)	210 (25.1)		
	Three or more times a week	68 (53.1)	60 (46.9)	128 (15.2)		

Table 6 demonstrates the relationship between depression and other behavioral factors. Internet addiction was measured by the Chen Internet Addiction scale and internet addiction was found to be significantly statistically associated with depression (χ ^2^ = 61.1, df 1, p = .000). Facebook addiction also had a significant relationship with depression among the sample population (χ ^2^ = 50.59, df1, p = .000). According to the data, nearly half of the sample medical students who were addicted to Facebook were also depressed, and this proportion was more than double when compared to those who were not addicted to Facebook. More than three-quarters of medical students who reported severe anxiety and high levels of perceived stress also reported depression, and the link between anxiety and depression, as well as the link between perceived stress and depression, was found to be statistically significant. (χ ^2^ = 273.32, df3, p = .000) and (χ ^2^ = 129.18, df2, p = .000). ^90^

**Table 6.** Relationship of depression with Internet addiction, Facebook addiction, anxiety, perceived stress, sleep quality.

Behavioral psychometric variables		<10 and ≥10		Total n (column %)	χ ^2^	p
		No depression n (row %)	Depression n (row %)			
Internet addiction	No addiction (<64)	461 (79.3)	120 (20.7)	581 (69.2)	61.1	.000
	Internet addiction (≥64)	137 (52.9)	122 (47.1)	259 (30.8)		
Facebook addiction	No Facebook addiction (<4)	468 (78.3)	130 (21.7)	598 (71.2)	50.59	.000
	Facebook addiction (≥4)	130 (53.7)	112 (46.3)	242 (28.8)		
Anxiety	Minimal	283 (95.3)	14 (4.7)	297 (55.4)	273.32	.000
	Mild	243 (76.2)	76 (23.8)	319 (38)		
	Moderate	66 (40)	99 (60)	165 (19.6)		
	Severe	6 (10.2)	53 (89.8)	59 (7)		
Perceived stress	(0-13) Low perceived stress	115 (94.3)	7(5.7)	122 (14.5)	129.18	.000
	(14-26) Moderate perceived stress	462 (73.3)	168(26.7)	630 (75)		
	(27-40) High perceived stress	21 (23.9)	67 (76.1)	88 (10.5)		
Pittsburg sleep quality index	Poor sleep	496 (68.6)	227(31.4)	723 (86.1)	16.94	.000
	Good sleep	102 (87.2)	15 (12.8)	117 (13.9)		

Finally, sleep quality was observed to be statistically related to depression among respondents (2 = 16.94, df1, p = .000), with data revealing that more than a third of poor sleepers experienced depression during the study period.

Multiple regression was used to assess significant predictors (anxiety, perceived sleep, sleep quality, perceived stress, internet addiction, romantic relationship, academic performance, sports and games and period of social media use) for depression score (PHQ9), after controlling for the influence of multiple sociodemographic and behavioral factors in the sample population. Preliminary analyses were performed to ensure that the assumptions of normality, linearity, multicollinearity, and homoscedasticity were not violated.

Total variance explained by the model as a whole was 59% and F change for the model was (26, 813) = 47.42, p < .001. In the model, anxiety, perceived sleep quality, sleep quality index, perceived stress, internet addiction, romantic relationship, academic performance, sports and games and period of social media use were found to be statistically significant predictors for depression (PHQ9 total score) yielding beta value (beta = .55, p = .000), (beta = .08, p = .001), (beta = .075, p = .007), (beta = .104, p = .001), (beta = .137, p = .000), (beta =.054, p = .028), (beta = -.071, p = .009), (beta = -.057, p = .012), (beta = - .077, p = .001) respectively (see Table 7).

**Table 7.** Depression predictors using a linear regression model.

	Standardized coefficients	t	Sig.	95.0% Confidence Interval for B	
	Beta			Lower bound	Upper bound
GAD 7 total score [anxiety]	.548	18.055	.000	.524	.651
Perceived sleep quality [very bad]	.081	3.386	.001	.771	2.896
Pittsburgh Sleep Quality Index (PSQI)	.075	2.702	.007	.037	.236
PSS 10 total score [stress scale]	.104	3.444	.001	.039	.144
CIAS total score [internet addiction scale]	.137	4.383	.000	.025	.065
Negative romantic relation	.054	2.202	.028	.097	1.682
Positive academic performance	-.071	-2.627	.009	-1.388	-.201
Sports and Games	-.057	-2.505	.012	-.992	-.120
How long have you been using social media	-.077	-3.284	.001	-.318	-.080

Dependent variable: Depression Model summary [R = .776, R square = .603, Adjusted R square = .59, F change = 47.42 (df1 26, df2 813), Sig. F change (.000)].

## Discussion

The purpose of this cross-sectional study was to determine the prevalence of depression among medical students, as well as potential depression predictors such as anxiety, perceived stress, internet addiction, Facebook addiction, sleep quality, sociodemographic and behavioral characteristics of medical students.

Depression was observed in 28.8% of medical students in our study, which is five times higher than the national prevalence of depression among adults. ^2^ The exact prevalence had also been observed among medical students in Thailand. ^28^ A meta-analysis of 77 studies that included 62,728 medical students and 1,845 non-medical students revealed a 28.0% global prevalence of depression among medical students. ^19^ Similar findings have been reported among medical students in Nepal, ^3^ Massachusetts ^4^ and Estonia ^39^ though Zaman *et al.* reported 39.1% depression in a previous study in Bangladesh ^53^ and Iqbal *et al.* observed 51.3% depression among Indian medical students. ^43^

In the current study, perceived stress was measured using PSS10B, and 85% of the medical students reported moderate to severe stress; however, in other DASS-based studies, 53% of medical students in India, ^43^ 63.7% in Egypt, ^73^ and 47.1% in Brazil ^74^ reported stress. A comparative study of public and private medical students in Bangladesh used GHQ-12 to measure stress, and the overall prevalence of stress was found to be 54%. ^75^ Because it is more about their feelings about lack of control and unpredictability than actual stressors, the prevalence of perceived stress may be higher than actual stress.

According to our findings, 65% of respondents experienced mild to severe anxiety, which is nearly double the result of a meta-analysis of 69 studies involving 40,348 medical students, which found that the overall prevalence of anxiety was 33.8%, ^76^ but is consistent with the findings of similar studies conducted in India ^43^ and Egypt. ^73^ However, anxiety was found to be most prevalent among medical students from the Middle East and Asia in a meta-analysis on the global prevalence of anxiety among medical students. ^76^

Internet addiction was found in 30.8% of medical students in our study, which is slightly higher than in two previous studies in Bangladesh. ^77^^,^^78^ The current rate (30.8%) was lower than in other Middle Eastern countries such as Jordan (40%) ^79^ and Iran (39.6%), ^80^ but higher than studies conducted among British (18.3%) ^81^ and Taiwanese (17.4%). ^82^ According to a systematic review, the prevalence of Internet overuse/possible Internet addiction among Southeast Asian students ranged from 7.4% to 46.4%. ^83^

Facebook addiction was found to be 28.8% in this study, which is 10% lower than a previous study in Bangladesh. ^29^

A recent study found that 69.5% of Bangladeshi medical college students had poor sleep quality, ^84^ whereas our data revealed that 86% of the study sample had poor sleep quality.

According to an editor’s note depression and anxiety are highly comorbid and their symptoms are frequently inseparable. ^85^ Previous research has also found that poor sleep quality, ^86^^–^^88^ poor academic performance, ^28^^,^^89^ and relationship status ^89^ are strong predictors for depression. Several studies reported a link between perceived stress and the presence of depressive symptoms, particularly severe depression. ^86^^,^^87^ Depression was 1.58 times more likely in the possible Internet addiction group than in the normal Internet use group, ^28^ and our data analysis confirmed this significant relationship. In the current study, anxiety, perceived sleep quality, sleep quality index, perceived stress, internet addiction, romantic relationship, academic performance, sports and games, and duration of having a social media account were all found to be statistically significant predictors of depression.

### Strengths and limitations

A strength of the study is that it was carried out with validated tools and a random sampling method. Because the current study is cross-sectional, data on the temporality of the relationship between variables is missing. Another flaw is that the mental health outcomes of medical students were not compared to clinician diagnoses.

## Conclusions

According to the current study, an alarming proportion of medical students suffer from depression. There is a strong link between depression and anxiety, stress, poor sleep quality, poor academic performance, and a negative social and romantic relationship status. Screening for depression and its associated factors among medical students should be prioritized in order to prevent and treat depression.

## Data availability

### Underlying data

Mendeley: Behavioral and psychosocial predictors of depression in Bangladeshi medical students: a cross-sectional study. doi: https://doi.org/10.17632/ykmywfnbbf.1 ^90^

This project contains the following underlying data:

- Depression among medical students.sav
- Code book for depression among medical students.spv

### Extended data

This project contains the following extended data:

- Depression among medical students English questionnaire.pdf
- Depression among medical students Bengali Questionnaire.pdf

Data are available under the terms of the Creative Commons Attribution 4.0 International license (CC-BY 4.0).
