# Supplementary material for: Behavioral and psychosocial predictors of depression in Bangladeshi medical students: a cross-sectional study
Source: F1000Res. 2022 Jul 5;11:745. [Version 1] doi: 10.12688/f1000research.122927.1 (PMC10904939; doi:10.12688/f1000research.122927.1)
Supplement: Peer review history

# Reviewer report: Reviewer response for version 1

**DOI:** 10.5256/f1000research.134978.r238190

**Published:** 2024-02-15

**Authors:** Hanaa E Bayomy

**Affiliations:** 1 Public Health & Community Medicine, Faculty of medicine, Benha University, Banha, Egypt

**Overall**, this manuscript is technically sound. The components of the research are represented appropriately, as regards the title, introduction, methods, results, discussion, and conclusion.

**Topic & title**: The manuscript introduces an important topic; depression among medical students that warrants careful examination. Medical students are at greater risk of depression due to workload, academic pressure, the pressure to demonstrate competence as a clinician, sleep deprivation, peer competition, fear of failure, the death and suffering of patients, student abuse, financial problems, etc. Depression can negatively impact students’ life quality, academic performance, and social relations. Thus, it is important to investigate this problem to determine predisposing factors and guide prevention and control interventions. The title of the study described the topic well and indicated the study design.

**Abstract**: A structured abstract is there with items of an abstract (background, methods, results, and conclusion). the keywords are suitable for the topic of the study, however, ‘medical students’ should be added to keywords to specify the study population.

**Introduction**: The topic of the study was defined and analyzed leading to the rationale and objectives of the study.

**Methods:** This is a cross-sectional study, suitable to assess the prevalence of depression in medical students. The study population comprised a random sample of medical students from four different medical colleges. Ethical considerations were cleared before implementing the survey and formal permission from the IRB was obtained as well as written consent from the participants. Validated instruments were used for data collection. Data collection instruments were carefully described and validated, as appropriate. Data are available from the given citation. Suitable statistical methods were used to test the validity of data collection tools such as Principal Component Factor Analysis and Cronbach’s Alpha, as well as to evaluate the association between depression and potential risks.

**Results**:

 Findings were presented in suitable tables and figures.

**Discussion**: Results were explained and discussed.

**Conclusion**: Coincided conclusion based on study results was mentioned.

**References**: Relevant Up-to-date references are presented in a systematic method.

Is the work clearly and accurately presented and does it cite the current literature?

Yes

If applicable, is the statistical analysis and its interpretation appropriate?

Yes

Are all the source data underlying the results available to ensure full reproducibility?

Yes

Is the study design appropriate and is the work technically sound?

Yes

Are the conclusions drawn adequately supported by the results?

Yes

Are sufficient details of methods and analysis provided to allow replication by others?

Yes

Reviewer Expertise:

Public Health and Community Medicine, Health Promotion, Preventive Medicine, and Epidemiology.

I confirm that I have read this submission and believe that I have an appropriate level of expertise to confirm that it is of an acceptable scientific standard.

# Reviewer report: Reviewer response for version 1

**DOI:** 10.5256/f1000research.134978.r185702

**Published:** 2023-07-28

**Authors:** Gerasimos N. Konstantinou

**Affiliations:** 1 Centre of Addiction and Mental Health, Toronto, Canada

Thank you for inviting me to review this very well written and interesting (as well as alarming) manuscript. It is indeed well known that medical students frequently have to face or deal with psychological difficulties during Medical School mainly due to the workload, academic pressure, pressure to demonstrate competence as clinicians, sleep deprivation, peer competition. As a result, it is anticipated that this population would experience increased levels of stress, perceived stress, depressive and anxiety symptomatology.

 The authors conducted a very well structured study and the were able to confirm this hypothesis. The language of the manuscript, as well as the structure of the manuscript and the references are appropriate. The authors may want to elaborate more on their findings in the discussion part, instead of just re-presenting their results, and they could potentially make some recommendations for future research or appropriate interventions that could/should be considered to protect/help this population. It would also be interesting to compare their result with those of countries in Europe, or North and South America, if any. Finally, I think the authors should also comment on the fact that the study was conducted in 2021, during the pandemic, which could be an additional component/stressor.

Is the work clearly and accurately presented and does it cite the current literature?

Yes

If applicable, is the statistical analysis and its interpretation appropriate?

Yes

Are all the source data underlying the results available to ensure full reproducibility?

Yes

Is the study design appropriate and is the work technically sound?

Yes

Are the conclusions drawn adequately supported by the results?

Yes

Are sufficient details of methods and analysis provided to allow replication by others?

Yes

Reviewer Expertise:

Clinical Psychiatry, Psychopathology, Brain Stimulation, Psychoimmunology

I confirm that I have read this submission and believe that I have an appropriate level of expertise to confirm that it is of an acceptable scientific standard.
